# Patient‐specific quality assurance using machine log files analysis for stereotactic body radiation therapy (SBRT)

**DOI:** 10.1002/acm2.13053

**Published:** 2020-10-19

**Authors:** Vivian U. Y. Chow, Monica W. K. Kan, Anthony T. C. Chan

**Affiliations:** ^1^ Department of Clinical Oncology Prince of Wales Hospital Hong Kong SAR China; ^2^ Department of Clinical Oncology The Chinese University of Hong Kong Hong Kong SAR China

**Keywords:** patient‐specific QA, log file analysis, stereotactic body radiation therapy, volumetric‐modulated arc therapy, plan delivery accuracy

## Abstract

An in‐house trajectory log analysis program (LOGQA) was developed to evaluate the delivery accuracy of volumetric‐modulated arc therapy (VMAT) for stereotactic body radiation therapy (SBRT). Methods have been established in LOGQA to provide analysis on dose indices, gantry angles, and multi‐leaf collimator (MLC) positions. Between March 2019 and May 2020, 120 VMAT SBRT plans of various treatment sites using flattening filter‐free (FFF) mode were evaluated using both LOGQA and phantom measurements. Gantry angles, dose indices, and MLC positions were extracted from log and compared with each plan. Integrated transient fluence map (ITFM) was reconstructed from log to examine the deviation of delivered fluence against the planned one. Average correlation coefficient of dose index versus gantry angle and ITFM for all patients were 1.0000, indicating that the delivered beam parameters were in good agreement with planned values. Maximum deviation of gantry angles and monitor units (MU) of all patients were less than 0.2 degree and 0.03 % respectively. Regarding MLC positions, maximum and root‐mean‐square (RMS) deviations from planned values were less than 0.6 mm and 0.3 mm respectively, indicating that MLC positions during delivery followed planned values in precise manner. Results of LOGQA were consistent with measurement, where all gamma‐index passing rates were larger than 95 %, with 2 %/2 mm criteria. Three types of intentional errors were introduced to patient plan for software validation. LOGQA was found to recognize the introduced errors of MLC positions, gantry angles, and dose indices with magnitudes of 1 mm, 1 degree, and 5 %, respectively, which were masked in phantom measurement. LOGQA was demonstrated to have the potential to reduce or even replace patient‐specific QA measurements for SBRT plan delivery provided that the frequency and amount of measurement‐based machine‐specific QA can be increased to ensure the log files record real values of machine parameters.

## 1 INTRODUCTION

Volumetric‐modulated arc therapy (VMAT) for stereotactic body radiation therapy (SBRT) can deliver high doses to target volumes while sparing proximal organs at risk (OARs) by generating a rapid fall‐off of dose outside the target in a hypofractionated regimen.^1^ The steep dose gradients are enabled by simultaneous modulation of multileaf collimator (MLC) positions, gantry rotation speeds, and dose rates during single or multiple gantry rotations around a patient.^2^

Conformal dose distribution can be generated by beam modulation in treatment planning system. However, extensive modulations utilized in SBRT can lead to deviation of delivered dose distribution from the planned one. This can be due to delivery system uncertainties such as MLC leaf position errors and gantry rotational instability.^1^ Therefore, pretreatment quality assurance (QA) of modulated arc therapy is necessary for patient safety.

According to American Association of Physicists in Medicine (AAPM) Task Group No. 218, measurement‐based patient‐specific QA methods are widely used and are the core element of most QA programs.^3^ A treatment plan with MLC leaf sequence file is computed on a homogeneous phantom with dosimeters to calculate the dose in QA geometry. The physical phantom is then irradiated to measure the actual dose distribution. Measured dose distribution can then be compared with the calculated dose distribution.^4^

Although measurement‐based patient‐specific QA is commonly used clinically, previous studies have demonstrated the insensitivity of this method to the discrepancy of beam parameters such as gantry angle errors and MLC positioning errors during data transfer or machine delivery.^5^ Therefore, analysis of machine log files has been suggested by several studies as an alternative.^6^, ^7^, ^8^ It can identify the problems that are undetectable with measurement‐based approach such as MLC positioning error. This is important as Budgell, et al. have demonstrated that accurate delivery of dose for IMRT fields require better than 1 mm leaf positioning accuracy.^8^, ^9^, ^10^, ^11^

It has been shown by Agnew, et al. that occasionally log file analysis might not be able to detect errors in MLC positions due to the failure of t‐nut or motor. However, it could still be a powerful tool to ensure data transfer accuracy associated with MLC performance, provided that more frequent measurement‐based machine‐specific QA has been established.^12^

Several studies have been performed to evaluate the efficiency and effectiveness of machine log file analysis for volumetric‐modulated arc therapy (VMAT).^13^, ^14^ Chan et al. conducted a preliminary study on quantifying the deviations of monitor unit (MU) and gantry angles during delivery against the plan through log analysis for three head and neck and three prostate VMAT cases.^14^ Mcgarry et al. quantified the plan delivery accuracy in terms of log file‐derived MLC positioning error with single center‐specific plan and single standard plan for each participating center in VMAT audits.^13^ However, no comprehensive study with a large number of clinical cases has been conducted to evaluate simultaneously the deviations of MU, gantry angles, and MLC positions against the planned values using machine log files. The accuracy of all these beam parameters is essential to guarantee mechanical stability and dosimetrical accuracy.

Also, extensive study has not been performed to investigate the use of machine log analysis for VMAT SBRT plans using flattening filter‐free (FFF) mode for various treatment sites. Since SBRT is indicated for localized small tumor, minor deviation introduced in MLCs might affect the dose distribution to a more significant extent compared with other radiotherapy treatment techniques.^4^, ^15^ This study is therefore aimed to demonstrate the effectiveness of an in‐house developed trajectory log analysis program (LOGQA) for evaluating the delivery accuracy of VMAT SBRT plans comprehensively with the introduction of intentional errors for software validation.

## 2 METHODS

### 2.1 Treatment plans

In this study, 120 VMAT SBRT were planned and delivered using the TrueBeam^TM^ linear accelerator (LINAC) with Millennium 120‐leaf MLC (Varian Medical Systems, Palo Alto, CA, USA). Treatment sites and number of plans included in the study were: 9 abdomen plans, 26 liver plans, 48 lung plans, 6 pelvis plans, 24 prostate plans, and 7 spine plans. The average number of arcs used in the study was four. Eclipse^TM^ (Varian Oncology systems, Palo Alto, CA) treatment planning system was used to generate the SBRT plans. Anisotropic analytical algorithm (AAA, version 13.6.23, Varian Medical Systems, Palo Alto, CA, USA) was used for dose calculation with the photon optimization algorithm (PO, version 13.6.23, Varian Medical Systems, Palo Alto, CA, USA) for plan optimization. A sequence of control points defining the MLC leaf positions and dose indices as a function of gantry angles was generated.^16^

### 2.2 Pretreatment QA measurements

Each VMAT SBRT treatment plan was calculated for the ArcCHECK^TM^ phantom (Sun Nuclear Corporation, Melbourne, FL, USA) using the Eclipse^TM^ treatment planning system.^17^, ^18^, ^19^ ArcCHECK^TM^ was then irradiated to measure the dose distribution of each plan. At the same time, machine log files were generated automatically. Global gamma‐index analyses with gamma criteria of 2 % dose difference and 2 mm distance‐to‐agreement (DTA) were performed using the SNC software (Sun Nuclear Corporation, Melbourne, FL, USA).^20^ The software compared the measured dose distributions with the planned dose distributions generated from the Eclipse^TM^ system using a dose calculation grid size of 0.25 cm. Absolute doses were used and the points with doses less than 10 % of the maximum doses were excluded in the gamma‐index analysis. Passing rate of gamma‐index analysis with 95 % or above was recognized as a pass in verification based on our local practice.

### 2.3 Machine log files analysis

Gantry angles, dose indices, and MLC leaf positions at each control point were retrieved from machine log files captured during pretreatment QA measurements. The delivered beam parameters of TrueBeam LINAC were recorded every 20 ms in a machine log file on a network drive.^13^ Trajectory log analysis was then performed using the in‐house developed program (LOGQA) written using Matlab (version 2016a, Mathworks Inc., Natick, MA, USA), which compared the delivered beam parameters with the planned values defined in the DICOM‐RT plan files (digital imaging and communications in medicine).

Reconstruction of Integrated transient fluence map (ITFM) was also performed in LOGQA based on the beam on and off status and MLC positions extracted from log files. Delivered fluence map was computed and compared with the planned fluence map. To reconstruct the delivered ITFM, fluence maps between two consecutive control points (CPs) were generated by considering the time for which the pixel of interest was being shielded by MLCs. By the summation of fluence maps over all the control points, delivered ITFM could be obtained. Each pixel in the delivered fluence map was then subtracted from the corresponding pixel in planned fluence map to calculate pixel‐by‐pixel difference.

**Fig. 1:**
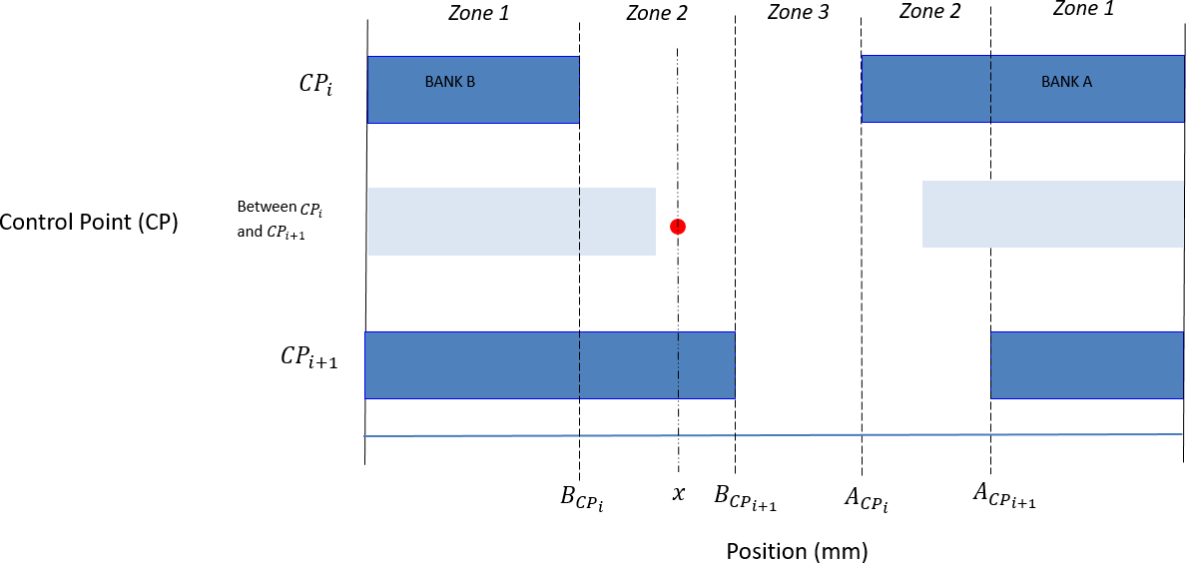
Reconstruction of fluence maps between two consecutive control points (CPs).

Each pixel in the fluence map was under one of the three zones illustrated in Fig. 1.

Zone 1) Shielded by the leaf all the time between two consecutive CPs. Only leaf transmission contributed to the fluence at the pixel of interest, which could be calculated by Eq. (1).

(1) $$\begin{array}{cc} \text{Fluence at any pixel in zone}\;1 & =\text{fractional MU between}\;2\;\text{CPs}\\ \;\times \;\text{Leaf transmission} \end{array}$$

Zone 2) Shielded by the leaf for a partial time between two consecutive CPs. Leaf transmission contributed to the fluence during the time portion when the pixel was shielded by MLCs, while fractional MU delivered between two CPs during the opening time contributed to the fluence when the pixel was unshielded by MLCs. Assume that the pixel of interest was at position *x* mm in Fig. 1 and its corresponding fluence could be calculated by Eq. (2).

(2) $$\begin{array}{cc} \text{Fluence at any pixel in zone}\;2 & =\text{Proportional of time being shielded}\;\\ & \;\times \;\text{fractional MU between}\;2\;\text{CPs}\\ & \;\times \;\text{Leaf transmission}\\ & \;+\;\text{Proportional of time being unshielded}\\ & \;\times \;\text{fractional MU between}\;2\;\text{CPs} \end{array}$$

where

$\text{proportional of time being shielded}=\frac{\left(B_{{\text{CP}}_{i+1}}‐\;x\right)}{\left(B_{{\text{CP}}_{i+1}}‐{\;B}_{{\text{CP}}_{i}}\right)}$ or $\frac{\left(x\;‐{\;A}_{{\text{CP}}_{i}}\right)}{\left(A_{{\text{CP}}_{i+1}}‐{\;A}_{{\text{CP}}_{i}}\right)}$

$\text{proportional of time being unshielded}=\frac{\left(x\;‐{\;B}_{{\text{CP}}_{i}}\right)}{\left(B_{{\text{CP}}_{i+1}}‐{\;B}_{{\text{CP}}_{i}}\right)}$ or $\frac{\left(A_{{\text{CP}}_{i+1}}‐\;x\right)}{\left(A_{{\text{CP}}_{i+1}}‐{\;A}_{{\text{CP}}_{i}}\right)}$

${\;A}_{{\text{CP}}_{i}}=\text{Position of Bank A MLC at control point}\;$ i [i = 1,2,……., Total number of CPs – 1].

${\;A}_{{\text{CP}}_{i+1}}=\text{Position of Bank A MLC at control point i}+1$ [i = 1,2,……., Total number of CPs – 1].

${\;B}_{{\text{CP}}_{i}}=\text{Position of Bank B MLC at control point i}$ [i = 1,2,……., Total number of CPs – 1].

${\;B}_{{\text{CP}}_{i+1}}=\text{Position of Bank B MLC at control point i}+1$ [i = 1,2,……., Total number of CPs – 1].

x = Position of pixel of interest

Zone 3) Unshielded by the leaf all the time between two consecutive CPs. Fluence was reconstructed by the fractional MU delivered between two CPs, which could be calculated by Eq. (3).

(3) Fluence at any pixel in zone3=fractional MU between2CPs

**Fig. 2:**
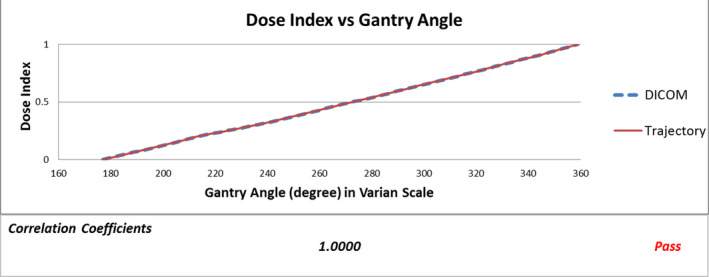
Dose index versus gantry angle.

**Fig. 3:**
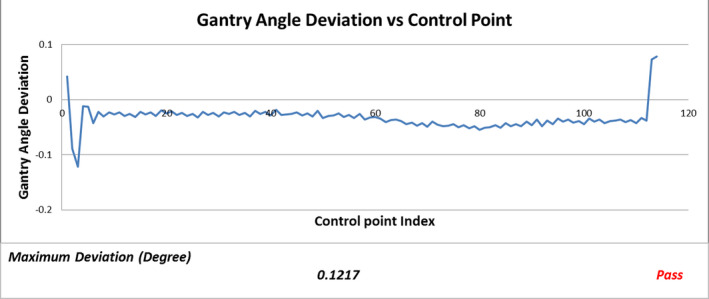
Gantry angle deviation versus control point.

**Fig. 4:**
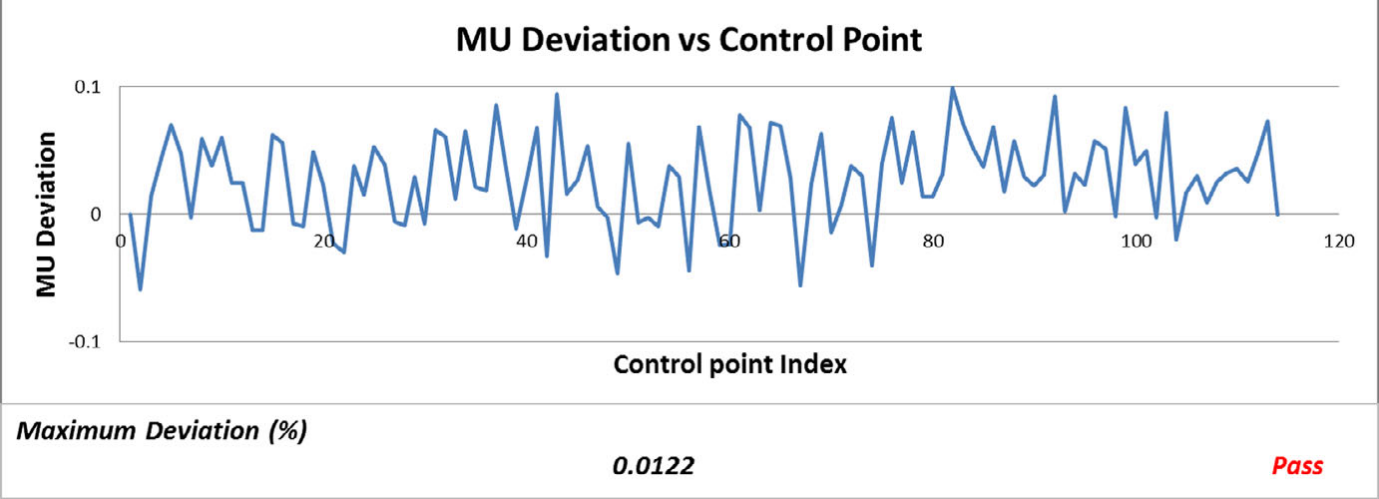
Monitor unit (MU) deviation versus control point.

**Fig. 5:**
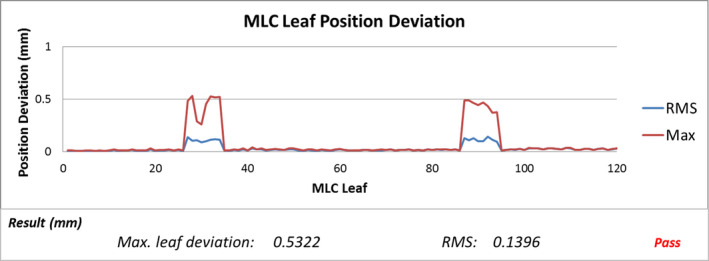
Multileaf collimator (MLC) leaf position deviation.

**Fig. 6:**
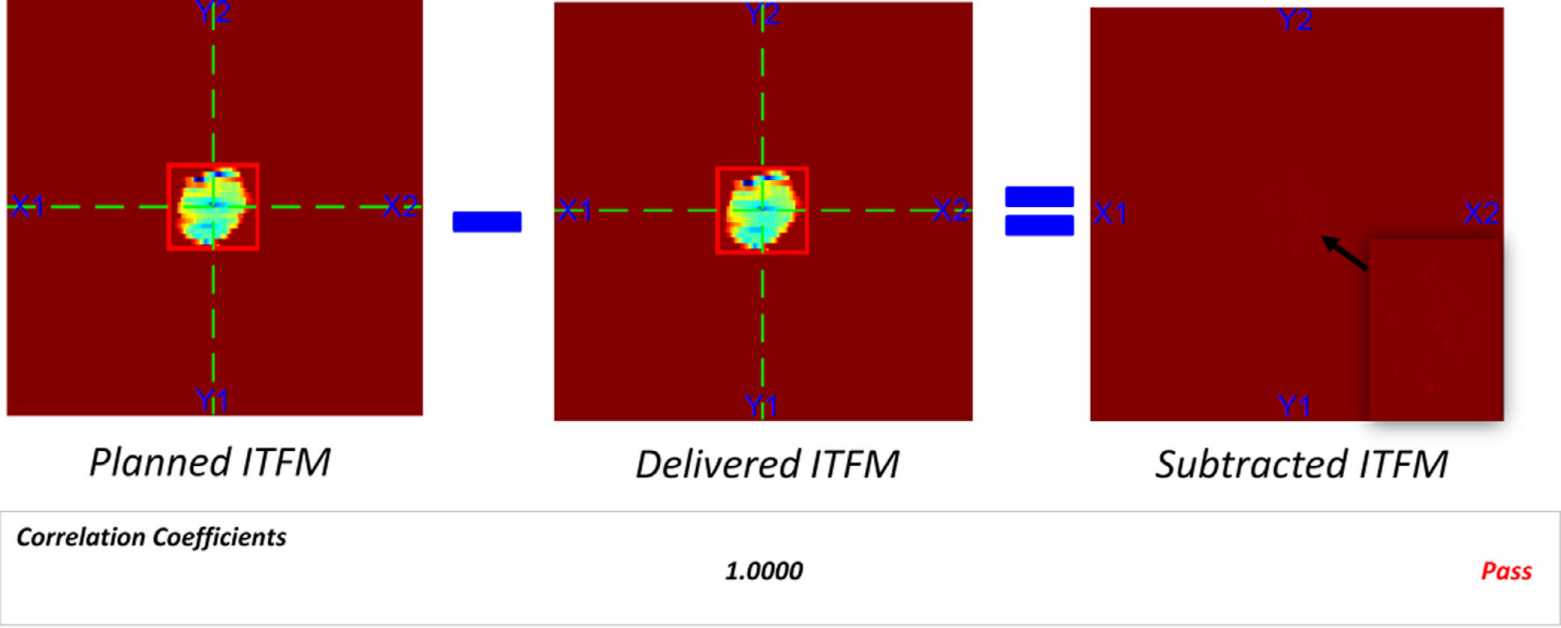
Planned and delivered integrated transient fluence map (ITFM) comparison.

Five graphs (Fig. 2, 3, 4, 5, 6) were plotted by LOGQA to demonstrate the accuracy of delivery for each plan, with quantitative indicators to define whether the comparison was passed or not (Table 1).

**Table 1:** Parameters for verifying the accuracy of plan delivery

Parameters to be checked by LOGQA	Quantitative Indicators with passing criteria
(1) Dose index (fractional monitor unit delivered) versus gantry angle	Correlation coefficient (CC) ≥0.985
(2) Gantry angle deviation versus control point	Maximum deviation ≤0.3 degree
(3) Monitor unit (MU) deviation versus control point	Maximum deviation ≤0.04 %
(4) Multileaf collimator (MLC) leaf position deviation	Maximum deviation ≤1 mm Root‐mean‐square (RMS) ≤0.5 mm
(5) Integrated transient fluence map (ITFM)	Correlation coefficient (CC) ≥0.985

Fig. 2 shows a sample of dose index versus gantry angle. Dose index refers to the fractional monitor unit delivered. Dose index values at different gantry angles for both planned values (blue dotted line) and extracted data from machine log (red solid line) are shown. Correlation coefficient is calculated to evaluate if the delivery is in good agreement with the plan.

Fig. 3 shows a sample of gantry angle deviation versus control point. Deviation of gantry angles at each control point against the planned value is calculated and shown on Fig. 2. Maximum deviation among the control point is compared with passing criteria (maximum deviation ≤ 0.3 degree).

Fig. 4 shows a sample of monitor unit (MU) deviation versus control point. Deviation of MU at each control point against the planned value is calculated and shown on Fig. 3. Maximum deviation of MU among all control points was normalized with the planned MU using Eq. (4) and compared with passing criteria (maximum deviation ≤0.04 %).

Fig. 5 shows a sample of multileaf collimator (MLC) leaf position deviation. Maximum deviation of MLC leaf positions among all control points (red solid line) and RMS (blue solid line) for each leaf were shown on Fig. 4 and compared with passing criteria (maximum deviation ≤1 mm and RMS ≤0.5 mm).

Fig. 6 shows a sample of planned and delivered integrated transient fluence map (ITFM). Reconstruction of delivered ITFM was based on beam on and off status and MLC positions extracted from machine log. Subtraction of delivered fluence from planned fluence was computed to calculate pixel‐by‐pixel difference. Correlation coefficient of planned and delivered fluence was also calculated to evaluate if the delivered fluence is in good agreement with the planned one.

(4) $$\text{Maximum MU deviation}\;\left(\%\right)=\frac{\text{Maximum deviation of MU}}{\text{Planned MU for the field}}\times 100\%$$

### 2.4 Software validation

Three types of intentional errors including MLC leaf positions, gantry angles, and dose indices were introduced to one patient plan for software validation.

- Leaf positioning errors of 1 mm and 3 mm in one of the leaf pairs (both leaf A and leaf B) for all the control points.
- Gantry angle errors of 1 degree and 2 degree for all the control points.
- Differential dose index errors of 5 % and 10 % for 60 control points.

Gamma criteria of 2 % dose difference and 2 mm distance‐to‐agreement (DTA) were used to compare the original plans and the log files of the plans with errors.

## 3 RESULTS

### 3.1 Evaluation of machine log files

Between March 2019 and May 2020, 120 VMAT SBRT treatment plans were evaluated using LOGQA and all the parameters being checked were within our tolerance listed in Table 1. Average correlation coefficient of dose index versus gantry angle and ITFM between the planned and delivered one for all the patients were 1.0000. Maximum deviation of gantry angle and MU of all patients were less than 0.2 degree and 0.03 % respectively. Regarding the MLC, the maximum deviation and root‐mean‐square (RMS) values were less than 0.6 mm and 0.3 mm respectively. Average deviation of MLC leaf positions, gantry angles and monitor unit from the planned values of various treatment sites was summarized in Table 2.

**Table 2:** Average error of MLC leaf positions, gantry angles, and monitor unit of 120 VMAT SBRT plans with various treatment sites

Treatment Site	MLC error (mm)	Gantry angle error (^0^)	Monitor unit error (%)
Abdomen	0.1318 ± 0.0184	0.1321 ± 0.0268	0.0152 ± 0.0075
Liver	0.1470 ± 0.0182	0.1263 ± 0.0127	0.0160 ± 0.0044
Lung	0.1445 ± 0.0200	0.1275 ± 0.0158	0.0142 ± 0.0022
Pelvis	0.1339 ± 0.0217	0.1287 ± 0.0222	0.0126 ± 0.0045
Prostate	0.1514 ± 0.0078	0.0999 ± 0.0165	0.0075 ± 0.0040
Spine	0.1276 ± 0.0112	0.0899 ± 0.0056	0.0063 ± 0.0012

### 3.2 Gamma‐index analysis of pretreatment measurements

Absolute gamma‐index analyses from ArcCheck^TM^ measurements were performed for each VMAT SBRT plan. The passing rate of all plans was larger than 95 % with 2 % dose difference and 2 mm spatial acceptance criteria, which would be recognized as a pass in verification based on our local practice.

### 3.3 Validation of LOGQA

**Fig. 7:**
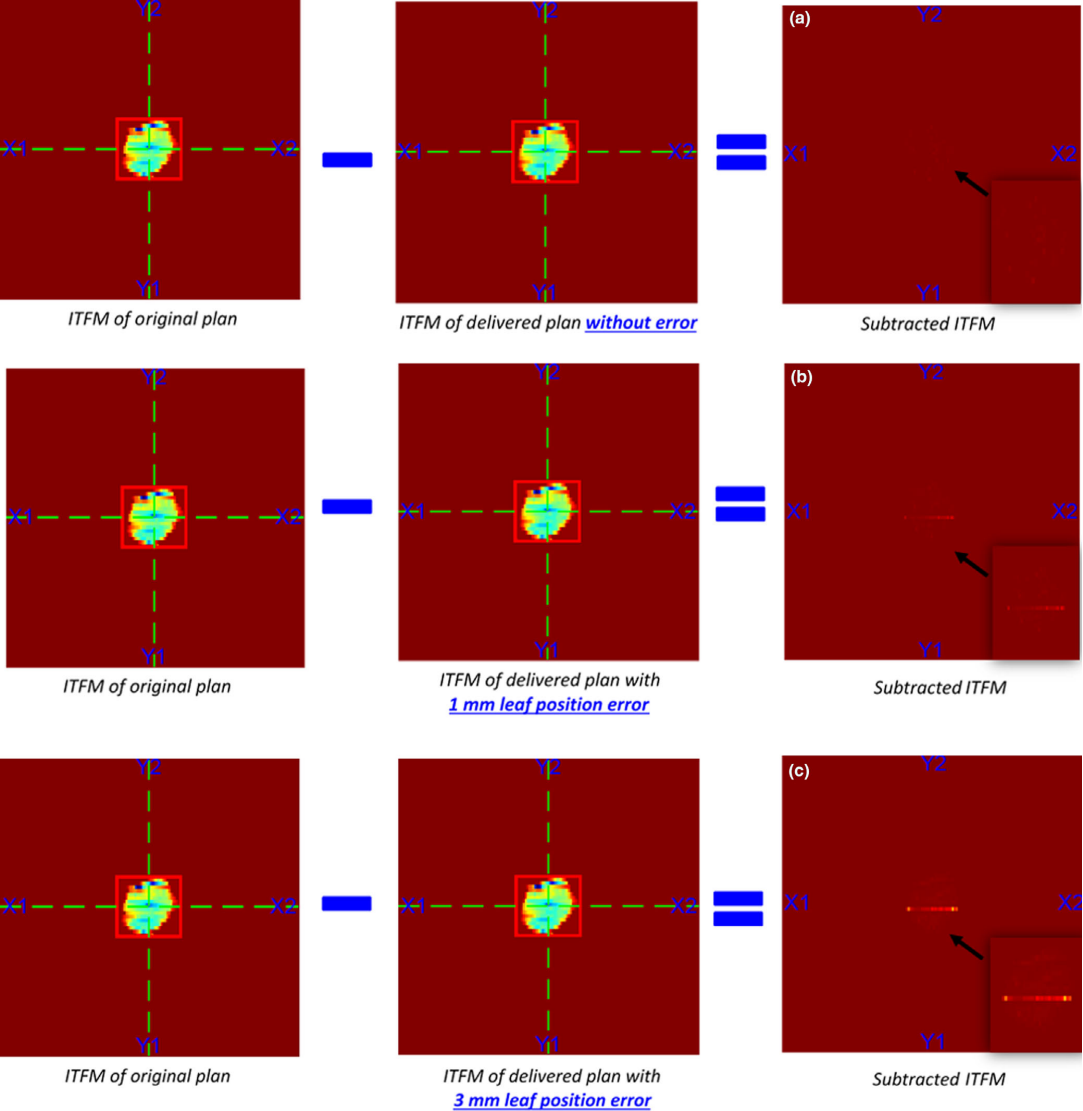
Result of LOGQA on ITFM analysis for a patient plan (a) without the introduction of intentional errors, (b) with an intentional MLC positioning error of 1 mm and (c) with an intentional MLC positioning error of 3 mm for all the control points of a leaf pair.

Intentional leaf positioning errors of 1 mm and 3 mm were introduced in one of the leaf pairs for all the control points in a patient plan. As shown in Table 3, LOGQA could figure out the 1 mm and 3 mm MLC leaf position deviations as failures according to the preset tolerance of LOGQA on maximum leaf deviation (≤1 mm) and RMS (≤0.5 mm). Also, residuals were visualized in subtracted ITFM for both the plans with 1 mm and 3 mm leaf position deviation in Fig. 7b and Fig. 7c respectively, compared with the original plan without the introduced error in Fig. 7a. The residuals were more clearly seen for the plan with 3 mm leaf error in Fig. 7c (3 mm error), compared with 7b (1 mm error). ArcCheck^TM^ measurement showed that with 1 mm leaf positioning error, the passing rate of gamma analysis was 96.9 %, which would still be recognized as a pass in verification based on our local practice (≥95 %). With 3 mm leaf positioning error, the passing rate decreased from 97.8 % to 92.7 %.

**Table 3:** Results of LOGQA and gamma analysis with the intentional leaf positioning errors of 1 mm and 3 mm introduced to a leaf pair of a patient plan for all the control points

	LOGQA						ArcCheck^TM^ measurement
	Maximum leaf deviation (mm) [Preset tolerance ≤ 1 mm]			RMS (mm) [Preset tolerance ≤ 0.5 mm]			Passing rate of gamma analysis (%) [Preset tolerance ≥ 95 %]
	Arc 1	Arc 2	Arc 3	Arc 1	Arc 2	Arc 3	
Original plan compared with delivered plan **without error**	0.5295 [Pass]	0.5263 [Pass]	0.5372 [Pass]	0.1267 [Pass]	0.1235 [Pass]	0.1201 [Pass]	97.8 [Pass]
Original plan compared with delivered plan with **1 mm leaf position errors**	1.5247 [Fail]	1.4450 [Fail]	1.5080 [Fail]	1.0252 [Fail]	1.0149 [Fail]	0.9521 [Fail]	96.9 [Pass]
Original plan compared with delivered plan with **3 mm leaf position errors**	3.5275 [Fail]	3.4450 [Fail]	3.5044 [Fail]	2.8983 [Fail]	2.7766 [Fail]	2.6747 [Fail]	92.7 [Fail]

In addition, gantry angle errors of 1 degree and 2 degree were introduced for all the control points in a patient plan. LOGQA could figure out the 1‐degree and 2‐degree gantry angle deviation as shown in Table 4. For ArcCheck^TM^ measurement, although the passing rate of gamma analysis decreased with the introduction of 1‐degree gantry angle error, the value was still higher than our local 95 % tolerance (95.8 %), which would still be recognized as a pass in verification. With 2‐degree leaf positioning error, the passing rate decreased from 97.8 % to 84.7 %.

**Table 4:** Results of LOGQA and gamma analysis with the introduction of 1‐degree and 2‐degree gantry angle errors in a patient plan for all the control points

	LOGQA			ArcCheck^TM^ measurement
	Maximum deviation (Degree) [Preset tolerance ≤ 0.3 degree]			Passing rate of gamma analysis (%) [Preset tolerance ≥ 95 %]
	Arc1	Arc2	Arc3	
Original plan compared with delivered plan **without error**	0.1120 [Pass]	0.0682 [Pass]	0.1149 [Pass]	97.8 [Pass]
Original plan compared with delivered plan with **1‐degree gantry angle error**	1.0749 [Fail]	1.1271 [Fail]	1.0748 [Fail]	95.8 [Pass]
Original plan compared with delivered plan with **2‐degree gantry angle error**	2.0812 [Fail]	2.1277 [Fail]	2.0815 [Fail]	84.7 [Fail]

Differential dose index errors of 5 % and 10 % were introduced for 60 control points in a patient plan. Maximum deviation of cumulative dose index reported by LOGQA increased significantly with increasing percentage of introduced error in differential dose index as shown in Table 5. That would indicate failures in the results of LOGQA for both the delivered plans with 5 % and 10 % differential dose index errors. For ArcCheck^TM^ measurement, the passing rate of gamma analysis decreased with the introduction of 5 % differential dose index error. However, it was still higher than our 95 % tolerance (97.1 %), which would still be recognized as a pass in verification according to local practice. With 10 % dose index error, the passing rate decreased from 97.8 % to 94.4 %.

**Table 5:** Results of LOGQA and gamma analysis with the introduction of 5 % and 10 % differential dose index errors in a patient plan for 60 control points

	LOGQA			ArcCheck^TM^ measurement
	Maximum deviation of cumulative dose index (%) [Preset tolerance ≤ 0.04 %]			Passing rate of gamma analysis (%) [Preset tolerance ≥ 95 %]
	Arc1	Arc2	Arc3	
Original plan compared with delivered plan **without error**	0.0132 [Pass]	0.0122 [Pass]	0.0124 [Pass]	97.8 [Pass]
Original plan compared with delivered plan with **5 % differential dose index error**	0.3776 [Fail]	0.3681 [Fail]	0.3787 [Fail]	97.1 [Pass]
Original plan compared with delivered plan with **10 % differential dose index error**	0.7674 [Fail]	0.7624 [Fail]	0.7794 [Fail]	94.4 [Fail]

## 4 DISCUSSION

Many potential errors can arise during treatment planning and delivery, such as inaccurate dose calculation and errors in plan transfer and delivery.^21^ There is a growing trend of performing machine log analysis for patient‐specific QA as it is more sensitive to identify mechanical errors of the order of 1 mm and 1 degree.^22^, ^23^ Also, machine log analysis can catch the discrepancies related to plan transfer and delivery problems.^24^, ^25^ In present study, 120 VMAT SBRT plans of various treatment sites were used to evaluate the delivery accuracy of plans through assessing the deviation of cumulative dose index, gantry angles, MLC positions and fluence with the use of machine log files.

Average correlation coefficient of dose index versus gantry angle and ITFM between the plan and delivery for all the patients analysed by LOGQA was 1.0000, indicating that the delivery is in good agreement with the plan. Maximum deviation of gantry angle and MU of all patients were less than 0.2 degree and 0.03 % respectively. Regarding the MLC positions, maximum deviation and root‐mean‐square (RMS) were less than 0.6 mm and 0.3 mm respectively, indicating that MLC positions during delivery followed the plan in a precise manner.

Magnitude of MLC errors (RMS <0.3 mm) and maximum gantry angle deviation (<0.2 degree) in this study for VMAT SBRT were similar to that reported for VMAT deliveries by Mcgarry, et al. (RMS <0.16 mm) and Chan, et al. (<0.1 degree) respectively.^13^, ^14^ Minor differences between studies might be due to the differences in the mechanical accuracy between machines such as the extent of degradation of MLC motors and wearing of gantry chain. To access the performance of MU delivery, percentage deviation of MU was calculated using Eq. (4) in this study instead of direct reporting of MU deviation as Chan et al.^14^ Since treatment plans with a range of MU were evaluated in present study, MU deviation was normalized with the planned MU for fair representation. The maximum percentage deviation of MU in this study was shown to be 0.03 %. Results of machine log analysis in this study were consistent with measurement‐based results, where the gamma‐index passing rate of all patients was larger than 95 %, with 2 % dose difference and 2 mm distance‐to‐agreement (DTA) criteria.

The developed LOGQA was also validated with known errors introduced in MLC leaf positions, gantry angles, and differential dose indices. LOGQA could successfully figure out the plans with errors of MLC positions, gantry angles, and differential dose indices with magnitudes of 1 mm, 1 degree, and 5 % respectively, while the passing rate of gamma analysis from ArcCheck^TM^ measurement was still higher than 95 % tolerance that would be recognized as a pass based on our local practice. Machine log analysis was demonstrated to be more sensitive in identifying small mechanical and dosimetrical discrepancies, which could be masked in composite patient dose measurement. Although with this advantage, trajectory log analysis could only be useful when the recorded parameters in log files reflect the real situation. As reported by Agnew et al, log file might not detect errors in MLC positions if there is failure in t‐nut or motor. This is due to the way the logs record the leaf positions as the number of turns performed by the motor. Leaf positions relative to motor might change in case a t‐nut is loose or broken, leading to wrong readout from motor that does not correspond to the real position of the leaf. It revealed the importance of a comprehensive measurement‐based machine‐specific QA program to ensure all machine parameters should be within tolerance if log files were used to reduce measurement‐based patient‐specific QA.^12^

Machine log files are useful in isolating errors during QA measurement. It helps to differentiate if the discrepancy is due to errors in TPS or errors in machine delivery. Besides VMAT SBRT, the use of LOGQA can extend to all the other modulated arc therapy plans. In addition to pretreatment QA, LOGQA can be used to monitor the delivery accuracy of each fraction of patient treatment. Any deviation in dose indices, gantry angles, and MLC positions during patients’ treatment can be identified and corrective actions can be implemented as early as possible. Also, degradation related to MLC and gantry can be spotted out earlier with the use of trajectory log analysis in a daily basis, thanks to its superior sensitivity in deviations of MLC leaf positions and gantry angles.^26^

## 5 CONCLUSION

Machine log analysis provides crucial information on VMAT SBRT plan delivery. End‐to‐end plan transfer and beam parameters accuracy check using LOGQA provides a robust and reliable QA method to reduce patient‐specific QA measurement, provided that a comprehensive measurement‐based machine‐specific QA program is in place. Good agreement of the trajectory plots and ITFMs between the delivery and the plan indicates precise modulation of dose rate, gantry rotational speed and MLC leaf speed, guaranteeing reliable mechanical stability and dosimetrical accuracy.

## CONFLICT OF INTEREST

No conflict of interest.
